# Influence of Polymer Modification on the Microstructure of Shielding Concrete

**DOI:** 10.3390/ma13030498

**Published:** 2020-01-21

**Authors:** Kamil Zalegowski, Tomasz Piotrowski, Andrzej Garbacz

**Affiliations:** Department of Building Materials Engineering, Faculty of Civil Engineering, Warsaw University of Technology, Al. Armii Ludowej 16, PL 00-637 Warsaw, Poland; t.piotrowski@il.pw.edu.pl (T.P.); a.garbacz@il.pw.edu.pl (A.G.)

**Keywords:** polymer modification, polymer dispersions, polymer fibers, concrete microstructure, image analysis method, stereological parameters

## Abstract

In this paper an analysis of the influence of polymer modification on the microstructure, shielding properties against neutrons, and compressive strength of heavy-weight magnetite concrete is carried out. The modifications involve the addition of acrylic or epoxy dispersions as well as micro- or/and macrofibers. A computer image analysis method is used to evaluate the microstructure of concretes and parameters of pore structure are calculated; these parameters include relative volume fraction, relative specific surface area, and pore arrangement ratios, including a proprietary ratio based on Voronoi tessellation. An assessment of significance of differences between stereological parameters of reference concrete and polymer modified concretes, as well as the impact of polymer form (dispersion or fibers) on shielding properties and compressive strength is carried out using Student’s *t*-test. The results show that except for the effect of the addition of both polypropylene micro- and macrofibers on the relative volume of pores, all other modifications result in statistically significant changes in the values of stereological parameters. Nevertheless, it is shown that neither polymer dispersions nor fibers have a statistically significant impact on shielding properties, but that they do influence compressive strength.

## 1. Introduction

The behavior of every material is related to its microstructure somehow. This is why understanding the relation between microstructure and properties is a basic approach of materials science and engineering. Hence, concrete design as a combination of a traditional approach based on relation composition-technology properties, with an analysis of microstructure, including searching for quantitative relations between microstructure and properties, could be the key to optimization of concrete’s technical properties.

The microstructure of concrete can be observed, registered, and analyzed at different levels, starting from the macroscopic through to the microscopic and nanometric scale, depending on the type of information to be obtained. Concrete microstructure encompasses a wide range of structural levels, from the atomic scale to that of the engineering macroscale. It may be composed of phases like aggregate grains, cement hydration products, unhydrated cement particles, fibers, and additions (e.g., microsilica, polymer dispersion, or waste materials [1,2,3]) as well as all discontinuities inside and between phases such as phase interfaces (e.g., the internal transition zone between aggregate grains and hardened cement matrix), pores, and cracks. The type, volume fraction, and arrangement of phases in the microstructure, the quality of their connection, and the characteristics of the pore structure vary depending on the quantitative and qualitative selection of the ingredients in the concrete mix. These, in turn, influence the mechanical performance, permeability of gases and liquids, and frost resistance, etc. [1,4,5,6].

It is believed that both quantitative and qualitative descriptions of processes and relations at a specific level should be sought at lower levels. Hence, seeking for relations between concrete properties and the microstructure seems to be reasonable. One of the basic relations between the microstructure and the properties of cement composites is the influence of porosity on strength [1,7,8]. It shows that an increase in pore volume fraction causes a decrease in compressive and tensile strength. But it is not the only factor, since the strength as well as durability is also determined by pore size and the uniformity of the arrangement of pores in the cement matrix [9,10,11]. It seems that the most favorable pores are those with the smallest dimensions which are uniformly arranged in the cement matrix. It could also be concluded that capillary pores and macropores significantly affect mechanical properties and the durability of cement composites, in contrast to smaller and inaccessible gel pores which do not contribute to liquid and gas transport.

The methods of microstructure analysis use different physical and chemical processes. Generally, they can be divided into microscopic technics, which enable the observations of microstructure morphology (i.e., sizes of the elements or their arrangement at different levels of magnification), and volumetric technics, which provide averaged information about the microstructure in a given volume of a material (e.g., thermogravimetry and X-ray diffraction). The group of volumetric techniques includes methods of testing the pore structure, e.g., mercury porosimetry, water or nitrogen absorption, and image analysis. Among these methods, only the image analysis method allows for testing the arrangement of pores apart from obtaining the volume fraction and specific surface area as well as the size distribution of pores, etc.

In this article the image analysis method is used to evaluate the microstructure of heavy-weight concretes modified by polymer addition in the form of dispersions or fibers. This type of concrete is recommended for shielding against ionizing radiation. Concrete has been applied as a shield against high-energy photons (gamma radiation) and neutrons since the beginning of the use of nuclear reactions in energy, medicine, and research. In the case of shielding against high-energy photons (gamma radiation) the increase in concrete density by change of aggregate type, e.g., from a normal to a heavy-weight type, is sufficiently effective [12,13]. Protection against neutrons is a more complex and sophisticated task [14,15,16], because neutrons are no-charge particles travelling in straight lines and interacting only with atomic nuclei while passing through matter. Neither the electrons surrounding a nucleus (the atomic electron cloud) nor the electric field caused by a positively charged nucleus affect a neutron’s flight. The type of this interaction depends mostly on the energy of moving neutrons. High energy neutrons (so-called fast neutrons) collide with nuclei, lose some of their energy as a result of elastic scattering, and change the direction of their movement. According to the laws of mechanics, the greater the mass of the nucleus, the smaller the share of energy which will be transmitted to it. Hence, the energy of neutrons is best scattered on light elements, like hydrogen. As the content of hydrogen in standard ordinary or heavy-weight concrete is rather small, in order to increase fast neutron attenuation, it must be increased. This can be done by modifying the composition of the concrete mix using materials containing hydrogen. One of the milestones in the evolution of shielding technology against neutron radiation was the concept of adding polymers into concrete composites in the form of admixtures (e.g., superplasticizers) and additives (e.g., resins and fibers) [17,18,19]. This enables w/c ratio (water-cement ratio) reduction and improvement of usability properties like tightness, mechanical properties, and chemical resistance without loss of the workability of the fresh concrete mix. In this study polypropylene micro- or/and macrofibers as well as acrylic or epoxy dispersions are used to increase the concentration of hydrogen atoms and to make attenuation of fast neutrons more effective. Other possible modifications of concrete composition leading to an increase in hydrogen content may involve addition of the special aggregate containing bound water (e.g., serpentinite or limonite).

When the energy of moving neutrons decreases to the appropriate level (that which is characteristic for so-called thermal or slow neutrons), the neutrons are absorbed by one of the nuclei of the atoms in the concrete. Otherwise, the neutrons leave the matter of the shield. As most concrete components are not the best absorbers, absorption efficiency should be improved by addition of some special components containing gadolinium or boron.

From the point of view of concrete modification, it is also important to note that some modifiers introduced into the concrete mix may cause a decrease in compression strength, which is the basic characteristic of concrete as a structural material [20].

The aim of this paper was to analyze how the polymer dispersions or fibers may influence the microstructure, effectiveness of neutron radiation shielding, and compressive strength.

## 2. Image Analysis

Image analysis is a type of stereological method; stereological methods are used for quantitative description of three-dimensional objects in a material volume based on measurements performed on two-dimensional images of a material microstructure (images of sample cross sections). The first attempts at applying this method in the construction sector took place at the beginning of the 1950s. These attempts involved comparing the observed microstructure with the scale of previously defined patterns in order to estimate grain size in metallic materials. They led to the development of one of the most known relations between the microstructure and properties which indicates that a reduction in average grain size causes an increase in tensile strength and hardness [21,22]. The development of computer techniques has made image analysis easier, faster, and more accurate. Over the years, the image analysis method has been employed in the study of a number of practical issues in the construction sector. This has been confirmed by the introduction of guidelines for characterizing pore structure in concrete in the EN 480-11 standard [23].

The investigation of the microstructure by the image analysis method is carried out on the surface of specially prepared samples which are called microsections. The key to obtaining correct and representative results is the precise taking of samples of a sufficient size whose surfaces then have to be prepared by grinding and polishing to ensure the representativeness of images that are taken of the microstructure. Preparation of the samples’ surfaces is preceded by sample mounting in a polymer resin in order to protect fragile materials or coatings, to distinguish pores from the rest of the microstructure constituents (if the polymer resin is colored), and to standardize sample sizes so they can be placed in a sample holder for grinding and polishing machines.

The images of the microstructure are commonly captured using light microscopes; however, it is also possible to use photo cameras or computer scanners [24]. The techniques listed above may be applied for evaluation of the microstructure at the micro- or macroscale, whereas the analysis of the microstructure at lower levels requires more sophisticated instruments, e.g., SEM or TEM [25].

**Figure 1:**
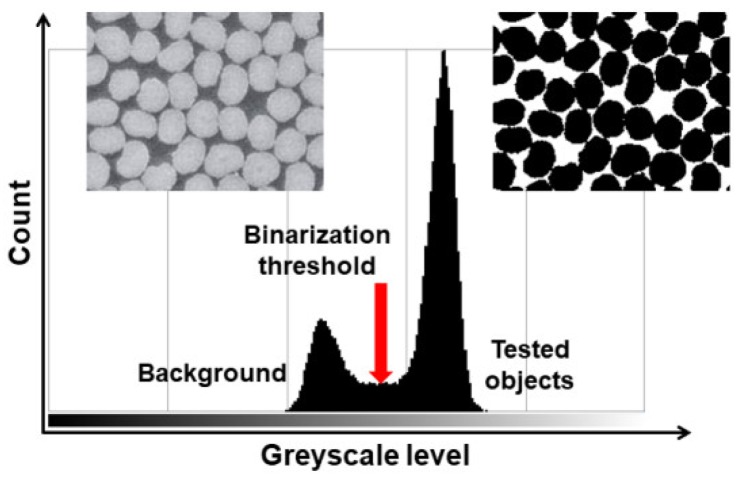
Scheme of image binarization with a global threshold: on the left is the output image and on the right is the image after segmentation.

The obtained images of the microstructure are first subjected to qualitative analysis in order to distinguish the constituents of the microstructure and to isolate those of them that are the subject of the image analysis by using digital image processing. Digital image processing is any modification of an image that changes the values of the RGB components (e.g., change of brightness or contrast, and binarization) or structure or form of objects on a binary image (i.e., morphological transformations, e.g., dilatation and erosion). The process of isolating a selected area of an image is called segmentation and it is performed on the basis of uniformity criteria, e.g., color, brightness level, and texture. The simplest means of image segmentation is binarization. This modification divides all the pixels into black pixels belonging to selected constituents of the microstructure and white pixels belonging to an insignificant background on the basis of a given brightness threshold (Figure 1).

The images of the microstructure after segmentation are subjected to quantitative analysis, mainly using computer programs that enable faster, more accurate, and more repeatable results than manual analysis. The binary image is the only type of image that programs which are currently used are able to analyze and unequivocally recognize various types of objects within; these objects are defined as coherent sets of points (usually black pixels) in which a transition between any two points is continuous without entering an area of different value (white pixels).

**Figure 2:**
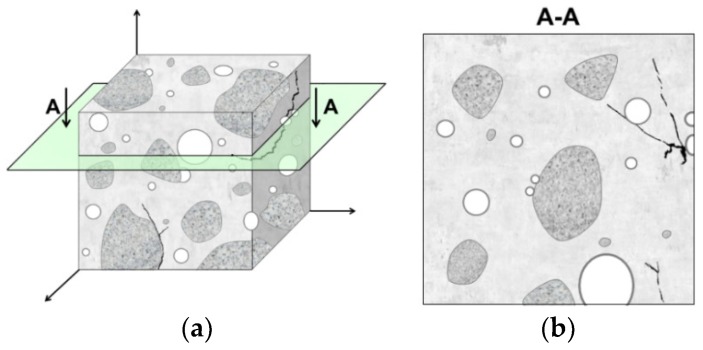
Hypothetical microstructure of cement composite containing aggregate grains, pores, and cracks: a) three-dimensional sample and b) two-dimensional cross-section of sample [24].

It should be noted that microstructure image capture causes a reduction in the number of dimensions of its constituents, i.e., three-dimensional objects like grains of aggregate or pores with a volume of *V* are represented by plane shapes with areas of *A*, interfacial transition zones between grains of aggregate and the hardened cement matrix are represented by curvilinear lines with length *L*, and cracks or fibers are likened to points *P* (Figure 2). On the basis of characteristics determined on the individual surfaces of sample cross sections (Table 1, parameters with index *A*), parameters describing the three-dimensional microstructure of a material are calculated (Table 1, parameters with index *V*). One of the basic stereological relations is the Cavalieri-Hacquert relation. It states that the volume fraction of selected objects (phase) in the volume unit of a material (*V_V_*) is equal to the surface area of selected objects in the image of the microstructure related to the area of the whole image, which is also equal to the number of points hitting selected objects in the image related to all points of the sample grid (Equation (1)). Another example is the Saltykov relation, which is used to calculate the relative surface area of selected objects in the volume unit of a material (*S_V_*). In the Saltykov method the image of the microstructure is pierced with a large number of vertical test lines with total length *L*; then, an expression is set up for the density of intersections with selected objects on the microstructure image (number of intersections *n*) (Equation (2)). The aforementioned equations are

(1) $$V_{V}=A_{A}=P_{P},$$

(2) $$S_{V}=2P_{L}=2\frac{n}{L},$$

where *V_V_* is the volume fraction of the selected objects in a volume unit of the material, *A_A_* is the surface area of objects related to the image area, *P_P_* is the number of points hitting selected objects related to all points of the sample grid, *S_V_* is the surface area of selected objects in a volume unit of a material, and *P_L_* is the number, *n*, of intersections of lines with a total length *L* with selected objects.

**Figure 3:**
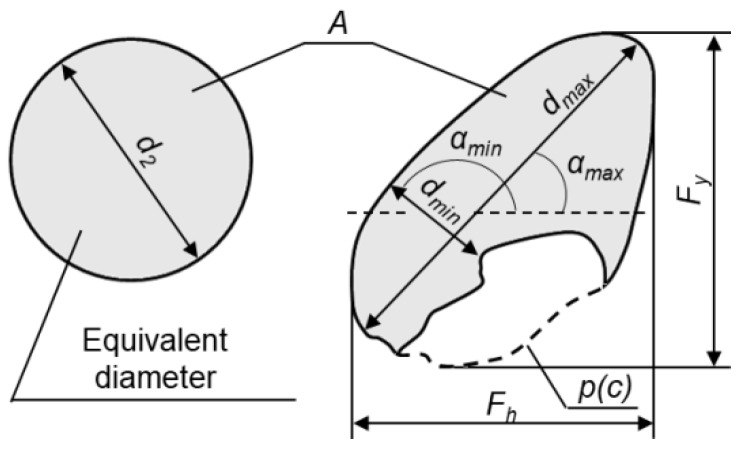
Basic parameters describing geometrical features of two-dimensional elements of a microstructure, e.g., grains of aggregates or pores. Legend: *d_2_*, equivalent diameter; *A*, area; *d_min_*, minimal size; *d_max_*, maximal size; *α_min_* and *α_max_*, orientation ratios; *p*(*c*), Cauchy perimeter; *F_h_* and *F_y_*, Feret diameters [24].

In the case of concrete, parameters which are commonly calculated describe the geometrical features of aggregate grains and pores, e.g., number, size, volume fraction, surface, shape, and arrangement (Figure 3) [26,27].

## 3. Materials and Methods

In this work concretes with heavy-weight aggregate and modified with polymer additives were studied. The reference concrete was a heavy-weight concrete with magnetite aggregate and slag cement CEM III/A 42.5N LH/HSR/NA (Lafarge, Małogoszcz, Poland). Aggregate mixtures contained fine aggregate (fraction 0/2 mm), which was river sand, and coarse magnetite aggregate with a maximum grain size of 16 mm (fraction 2/8 mm and 8/16 mm). The cement was characterized by an extended setting time, normal early compressive strength, high resistance to sulphate aggression, low hydration heat, and low alkali content. It was composed of Portland clinker (35–50%), granulated blast furnace slag (50–65%), and a setting time regulator, namely, calcium sulfate (5%). The reason for choosing this type of cement was the fact that the tested concretes were intended for shielding structures of reactors in nuclear power plants or research centers, which are usually massive structures and require a reduction in cement hydration heat to limit thermal stress.

The reference magnetite concrete *M* was modified with the addition of acrylic (*MPCC1*) or epoxy dispersion (*MPCC2*) as well as addition of polypropylene macro- (*MF1*) and/or microfibers (*MF2* and *MF3*). Acrylic dispersion involved a liquid containing carboxylate styrene-butadiene latex, which is generally used to modify mixes of concretes and mortars. Epoxy dispersion involved two-component water dispersion used mainly as a colorless impregnate and a concrete ground strengthener for concrete as well as a modifier of mortar mixes. The amount of both acrylic and epoxy dispersion addition was set at a level of 7.0% in relation to the weight of the cement considering only the weight of polymer itself, which was equal to about 47.0% of the dispersion mass. Two types of polypropylene fibers were used, namely, microfibers 12.0 mm in length and 0.038 mm in diameter and macrofibers 39.0 mm in length and 0.78 in diameter. The quantity of micro- and macrofibers used was equal to 0.6 and 3.5 kg per m^3^ concrete mix, respectively.

The water/cement ratio of the concrete mixtures was fixed to 0.4. Consistence was measured by the slump test method and kept at an S4 level (160–210 mm) by changing the amount of the superplasticizing admixture.

A detailed composition of the concretes used in the study is given in Table 2.

**Figure 4:**
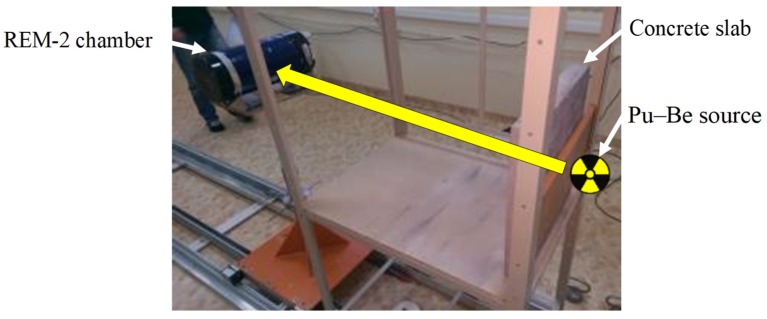
Experimental stand for measurements of neutron radiation shielding efficiency [28].

The investigation of the effectiveness of shielding against neutrons was measured using a Pu–Be source and a specially prepared stand that allowed placing 500 mm × 500 mm × 50 mm concrete slabs perpendicularly to the positioning mechanism of the radiation source (Figure 4). The measurements were made according to the individually developed program [18]. The evaluation was based on the half value layer (*HVL*) expressing the thickness of the absorbing material needed for a reduction in the incident radiation intensity by a factor of two. Using the appropriate recombination techniques, the neutron radiation dose equivalent was determined, and based on the neutron radiation dose equivalent reduction curves, one *HVL* per concrete was calculated. The details of the experiments on shielding effectiveness and other technical characteristics of tested concretes have been presented elsewhere [24].

A compressive strength test was carried out on 10 cm × 10 cm × 10 cm cubic samples at an age of 28 days. Compressive strength for each concrete was the mean value of results obtained for three samples.

**Figure 5:**
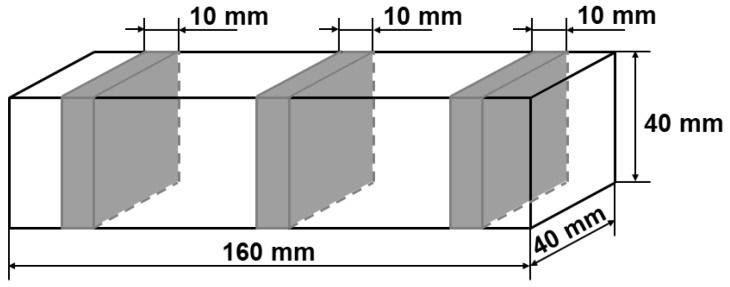
Scheme of sample cutting for computer image analysis.

**Figure 6:**
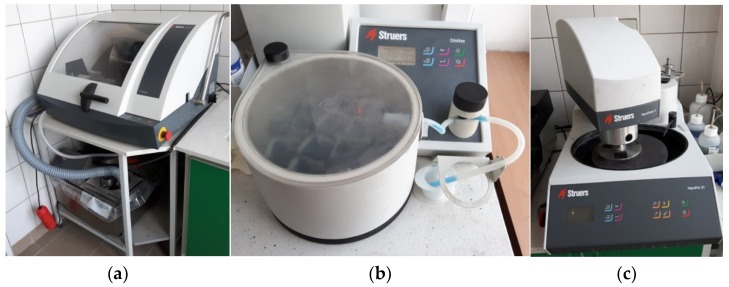
Equipment used in sample preparation for image analysis: (**a**) cutting machine (Labotom-5, Struers, Cleveland, OH, USA), (**b**) vacuum impregnation machine (CitoVac, Struers, Cleveland, OH, USA), and (**c**) polishing and grinding machine (Tegrapol-21, Struers, Cleveland, OH, USA).

**Figure 7:**
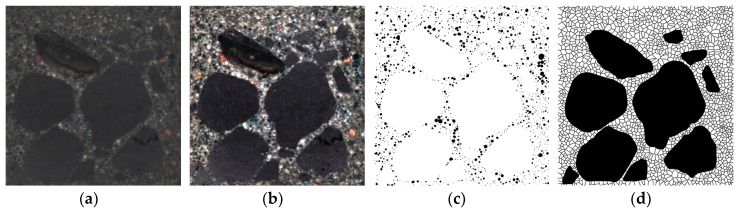
Scheme of image preparation for computer image tessellation: (**a**) received image, (**b**) processed image, (**c**) binarized image of pore structure, and (**d**) binary image of pore structure after tessellation with marked coarse aggregate grains (grains with dimensions above 4 mm).

The microstructure of the concretes was evaluated by computer image analysis. A set of three samples with the dimensions 40 mm × 40 mm × 160 mm was prepared for each concrete and cured for 28 days. The procedure of sample preparation for microstructure studies involved (1) cutting out three slices about 40 mm × 40 mm × 10 mm in size (Figure 5, Figure 6a) from three samples for each concrete (i.e., a total of nine slices for each concrete composition were produced), (2) cold mounting under lowered pressure using a colored epoxy resin (Figure 6b), (3) grinding, and (4) final polishing (Figure 6c). After this, 2D images of the microstructure with a resolution of 2400 DPI (1 pixel was equal to 10.6 µm) were taken using a computer scanner. The images were subjected to computer processing in order to obtain the most precise binary image of black pores, which were of interest, on a white background of other microstructure constituents. The image processing was a combination of changes of contrast, brightness, gamma modulation, and color saturation (Figure 7b), followed by manual selection of pores and binarization (Figure 7c). Quantitative analysis of the concrete microstructure was performed using a proprietary computer program working in a MATLAB environment which was developed at the Faculty of Civil Engineering of Warsaw University of Technology. This program was used to calculate relative the volume fraction of pores *V_V_*, the relative surface area *S_V_* and the ratio of pore arrangement $\bar{L}$ according to the EN 480-11 standard. Additionally, the authors proposed a method for evaluation of the uniformity of arrangement of pores based on Voronoi tessellation.

The ratio of pore arrangement $\bar{L}$ calculated according to the EN 480-11 standard is a parameter defining the maximum distance between any point of the hardened cement matrix in concrete and the edge of a pore. To calculate the $\bar{L}$ value the traverse method should be used. This method consists of conducting observations along a set of measurement lines parallel to the original top surface of the sample, followed by a determination of the number of intersections of these lines with pores and measurement of the length of chords passing through pores. The $\bar{L}$ parameter is calculated from one of two equations based on the ratio *R* of the volume of the cement matrix (calculated on the basis of the concrete mix recipe) to total air content. In the case of *R* > 4.342 Equation (3) is used, while for *R* ≤ 4.342 Equation (4) must be applied. Equation (3) and Equation (4) are

(3) $$\bar{L}=\frac{3\left[1,4{\left(1+R\right)}^{1/3}-1\right]}{\alpha }\left[\mathrm{mm}\right]$$

(4) $$\bar{L}=\frac{P\times T_{tot}}{400*N}\left[\mathrm{mm}\right]$$

where *R* is the ratio of the volume of the cement matrix and total air content, α is the specific surface area of pores, *P* is the volume of the cement matrix, *T_tot_* is the total length of measurement lines, and *N* is the total number of chords passing through the pores.

In the method proposed here for the evaluation of pore arrangement, the straight lines divide distances between pairs of closest pores (the centers of pores) in half, and the intersections of these lines determine the corners of polygons (in other words the image area is divided into smaller areas), which are so-called “impact zones” (see Figure 7d) which belong to particular objects. The microstructure is considered to be homogeneous in terms of the arrangement of given objects (e.g., pores) if they will be arranged in the material in such a way that each of them will have statistically the same neighborhood (coordination). Hence, the obtained mean equivalent diameter of “impact zones”, *d_2V_*, should be characterized by the lowest possible coefficient of variation *CV*(*d_2V_*). Voronoi tessellation of binary images of pore structure was also performed using MATLAB.

Stereological parameters calculated by computer image analysis were characterized by mean value $\bar{X}$ and coefficient of variation *CV*.

The influence of polymer addition on the microstructure of reference concrete as well as shielding against neutrons and compressive strength was determined using a mathematical statistics method, namely, Student’s *t*-test. In essence, this test compares differences in means of two sets of independent measurements, assuming normality of data and equality of variances across comparison groups. As a result, a *t* statistic and *p* value are calculated and compared with critical values of the *t* distribution for the appropriate number of degrees of freedom and defined significance level, respectively. If the value of the *t* statistic is sufficiently high and the *p* value is lower than the defined significance level, the difference between the means could be considered to be “statistically significant”, that is, that the underlying distributions very likely have different means. In the presented paper, for the purpose of calculations, the null hypothesis proposed that no statistical significance would exist between the means of the stereological parameters, shielding properties, and compressive strength of *M* concrete and other concretes (*MPCC1*, *MPCC2*, *MF1*, *MF2* and *MF3*); the alternative hypothesis was that the differences between the means would be significant, with the significance level being *p* = 0.05.

**Figure 8:**
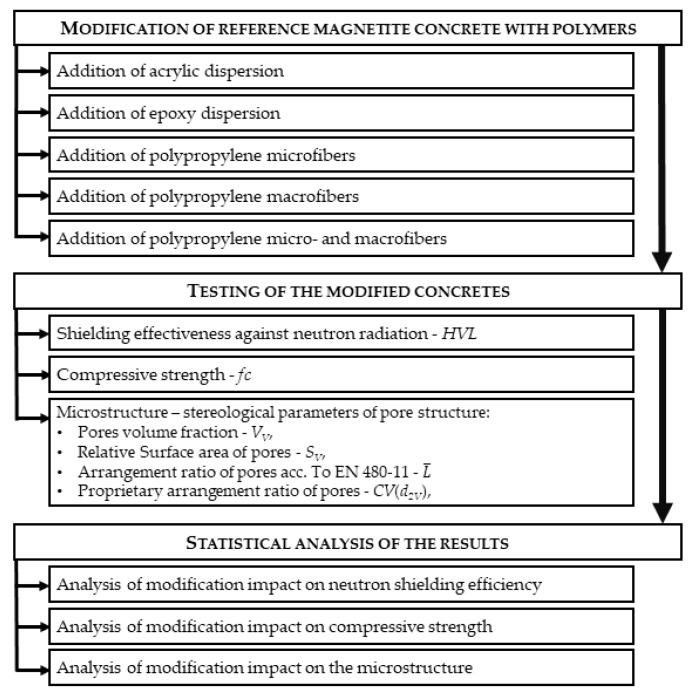
Scheme of the experimental program.

The details of the experimental program are presented in Figure 8 and a summary of the performed tests and number of samples used for each composition is given in Table 3.

## 4. Results and Discussion

### 4.1. Neutron Radiation Shielding Effectiveness

The experimental results of the shielding properties against neutrons are presented in Table 4. The modification of reference concrete *M* had a different influence on the *HVL*. The addition of acrylic dispersion (*MPCC1*) or macrofibers (*MF1*) to *M* had a minor effect on the *HVL*, namely, changes were only up to about 3.0%. On the other hand, it was observed that the epoxy dispersion (*MPCC2*), macrofibers (*MF2*), and both types of fibers added at the same time caused an increase in *HVL* from about 5.0% (*MPCC2*) to 17.0 (*MF3*); in other words, the thickness of the shield necessary to reduce the radiation by half increased, which means that the shielding effectiveness was weakened. This is not consistent with the results reported in the literature [17,18,19]. Because of the increase in hydrogen content, the addition of polymers to concrete should result in an increase in its effectiveness in shielding against neutron radiation. The explanation of this phenomenon requires broader study.

### 4.2. Compressive Strength

The results of the compressive strength test are presented in Table 5. The modified concrete *MPCC1* (with acrylic dispersion addition) and *MF1* (with polypropylene microfibers addition) showed compressive strength similar to reference concrete *M*, while *MPCC2* (with epoxy dispersion addition) and *MF2* (with polypropylene macrofiber addition) had about 21.0% higher strength than *M*. By contrast, the addition of both micro- and macrofibers at the same time (*MF3*) resulted in a large decrease in compressive strength of up to 40.0%. This was probably caused by adding too large an amount of fibers, leading to a drop in workability and to an increase in the dimensions of pores, which was proven using the image analysis method (Section 4.3). The literature confirms that low porosity is not the only condition which leads to good quality and durable concrete, since pore size distribution also plays an important role [15].

### 4.3. Stereological Parameters

The results of the computer image analysis are presented in Table 6. It can be observed that the addition of both acrylic (*MPCC1*) and epoxy dispersions (*MPCC2*) as well as micro- (*MF1*) and macrofibers (*MF2*) caused a decrease in the porosity *V_V_* of M from about 59% (*MPCC2*) up to 82% (*MPCC1*), while the addition of both types of fibers simultaneously (*MF3*) resulted in a minor increase in *V_V_* of about 9%. The decrease in *V_V_* after adding acrylic dispersion as well as epoxy dispersion to *M* was expected, since this type of polymer addition usually causes an increase in the tightness of the microstructure of cement composites, and thus an increase in durability. All performed modifications of *M* created a decrease in *S_V_* of *M* from 45% (addition of both micro- and macrofibers, *MF3*) up to 75% (acrylic dispersion, *MPCC1*). On the other hand, the additions used in the study induced an increase in the $\bar{L}$ of *M* from about 42% (addition of acrylic dispersion, *MPCC1*) up to 89% (addition of both micro- and macrofibers, *MF3*). Similar values of *V_V_* and radically different values of the $\bar{L}$ of M and *MF3* concrete prove that addition of both micro- and macrofibers (*MF3*) resulted in an increase in pore diameters in relation to the reference concrete (*M*). Moreover, it was shown that in contrast to the rest of the parameters, the influence of additions on the ratio of uniformity of pore arrangement *CV*(*d_2V_*) of *M* was relatively small, with the ratio increasing from about 4% (addition of both micro- and macrofibers, *MF3*) to 11% (addition of microfibers, *MF1*). This leads to the conclusion that the uniformity of pore arrangement in the concretes remained almost unchanged.

The variability of *V_V_* results was diversified and the largest variability characterized *V_V_* of magnetite concrete modified with epoxy dispersion (*MPCC2*, *CV*(*V_V_*) = 24.41%) due to the presence of macropores reaching a few millimeters in diameter and appearing in a completely random way and overstating the *V_V_* of some samples. A slightly lower *CV*(*V_V_*) was noticed in the case of *MF3* concrete modified by both micro- and macrofibers, and this heterogeneity of pore structure is not surprising because of the drop in the workability observed during the mix preparation, which caused difficulties with a compaction. The coefficients of variation *CV*(*S_V_*) and *CV*($\bar{L}$) of the tested concretes varied within a narrow range from 5.37% to 12.46%.

### 4.4. Analysis of Modification Impact on Neutron Shielding Efficiency

The results of analysis regarding the significance of differences between the *HVL* obtained for the reference concrete *M* and the two groups of concretes modified with polymer dispersions (*MPCC1* and *MPCC2*) or fibers (*MF1*, *MF2*, and *MF3*) are presented in Table 7. Neither polymer dispersions nor fibers had a statistically significant impact on *HVL* because the *t* statistics were not in the critical region: *K* = (−∞;−6.3138∪6.3138;+∞) or *K* = (−∞;−2.9200∪2.9200;+∞), respectively, and the *p* values were much higher than the defined significance level *p* = 0.05 as well. This meant that the null hypothesis was true (*H_0_*, i.e., no statistical significance existed between the means of stereological parameters). Taking into consideration the similar effect of both forms of polymer additions on the *HVL*, it could be concluded that concretes modified with polymer dispersions should be used for shielding purposes against ionizing radiation. This is because their mixes are easier to design and place than concrete mixes containing polymer fibers, which additionally may show a decrease in workability.

### 4.5. Analysis of Modification Impact on Compressive Strength

The results of analysis of the significance of differences between compressive strength, *fc*, obtained for *M* and two groups of concretes modified with polymer dispersion (*MPCC1* and *MPCC2*) or fibers (*MF1*, *MF2* and *MF3*) are presented in Table 8. In contrast to *HVL*, the *fc* was statistically significantly dependent on the form of polymer introduced into the reference concrete mix. The *t* statistics in this case were in the critical region: *K* = (−∞;−2.0150∪2.0150;+∞) or *K* =(−∞;−1.8595∪1.8595;+∞) for dispersion additions and fiber additions, respectively, and the *p* values were significantly lower than the defined significance level *p* = 0.05. Comparing the influence of additions of both forms of polymers, it can be observed that dispersions influenced *fc* in a more effective way; the *p* value for this group of concretes was one order of magnitude lower than for the group of concretes modified with fibers. It is possible to achieve almost the same effect (the compressive strengths of *MPCC1* and *MF1* and of *MPCC2* and *MF2* were similar) using polymer dispersions and fibers, so yet again, like in the case of the *HVL*, it is recommended to use concrete modified with polymer dispersions, not fibers.

### 4.6. Analysis of Modification Impact on the Microstructure

**Figure 9:**
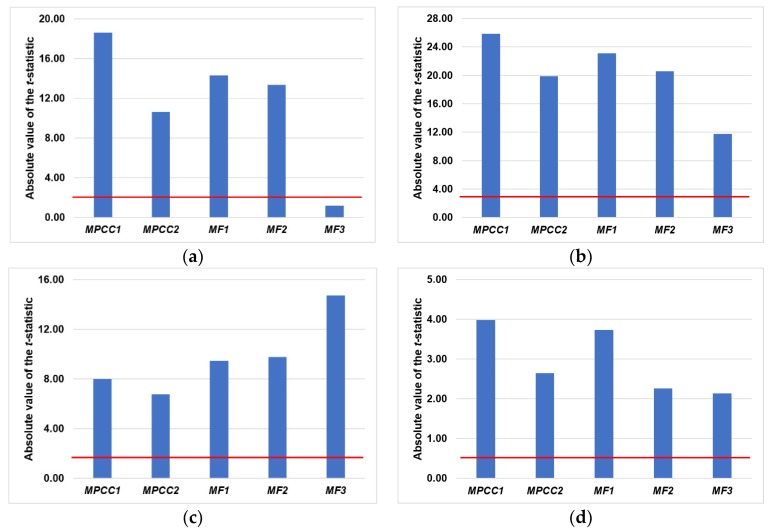
Results of Student’s *t*-test for stereological parameters: (**a**) *V_V_*; (**b**) *S_V_*; (**c**) $\bar{L}$; and (**d**) *CV*(*d_2V_*) (the red line shows the level of the defined significance level *p* = 0.05).

The results of analysis on the significance of differences between calculated stereological parameters are presented in Table A1 (see Appendix A) and Figure 9. Student’s *t*-test showed that for the defined significance level (*p* = 0.05), except for the effect of the addition of both micro- and macrofibers (*MF3*) on porosity *V_V_*, all performed modifications of the reference concrete resulted in statistically significant changes in the values of the stereological parameters. This is reflected in the *t* statistics values, which were almost entirely in the critical region *K* = (−∞;−1.8595∪1.8595;+∞) (Figure 9, Table A1 (see Appendix A)) and in the values of probability *p* (Table A1 (see Appendix A)), which were much lower than the limit value *p* = 0.05. Both the *t* statistics and the probability *p* values provided a solid basis for the rejection of the null hypothesis. In summary, the obtained results indicate that changes in the values of microstructural parameters which were introduced by polymer modification of the reference concrete mix are large enough to be considered as significant. It was also proven that the computer image analysis method is appropriate to use to differentiate the changes in the concrete microstructure caused by polymer additions which are used.

## 5. Conclusions

In this paper an analysis of the influence of polymer modification on the microstructure of heavy-weight magnetite concrete was carried out. The obtained results showed that the computer image analysis method is appropriate to use to differentiate the changes in the concrete microstructure introduced by modification with polymers. For the defined significance level (*p* = 0.05), except for the effect of the addition of both polypropylene micro- and macrofibers at the same time on a relative volume of pores, all modifications of the reference concrete resulted in statistically significant changes in the values of stereological parameters. Neither the polymer dispersions nor fibers had a statistically significant impact on *HVL*, but they influenced the compressive strength.

## Figures and Tables

**Table 1:** Basic parameters used to describe objects on a material cross section and within its volume.

Parameter	Definition
*A_A_*	Surface area of objects related to the image area
*L_A_*	Line lengths related to the image area
*N_A_*	Number of objects related to the image area
*V_V_*	Volume fraction of the selected objects in a volume unit of a material
*S_V_*	Surface area of the selected objects in a volume unit of a material
*L_V_*	Line lengths in a volume unit of a material
*N_V_*	Number of selected objects in a volume unit of a material

**Table 2:** Composition of concretes used in the study.

Short Name	Symbol	Definition								
		kg per m^3^ of Concrete Mix							% of C (mass %)	
		*C*	*W*	*S*	*MG*	*P*	*PP*	*SA*	*A*	*E*
Heavy-weight	*M*	380	152	750	2078	-	-	0.56	-	-
Heavy-weight with polymer	*MPCC1*	380	152	750	2078	-	-	0.26	10	-
	*MPCC2*	380	152	750	2078	-	-	0.43	-	10
Heavy-weight with polymer fibers	*MF1*	380	152	750	2078	-	0.6	1.03	-	-
	*MF2*	380	152	750	2078	3.5	-	0.52	-	-
	*MF3*	380	152	750	2078	3.5	0.6	0.52	-	-
**Legend:**										
*C*—CEM III/A 42.5N LH/HSR/NA *W*—water *S*—river sand		*MG*—crushed magnetite *A*—acrylic dispersion *E*—epoxy dispersion				*P*—polypropylene macrofibers *PP*—polypropylene microfibers *SA*—superplasticizing admixture				

**Table 3:** Summary of performed tests and the number of samples used for each composition.

Test	Sample Size (cm^3^)	No. of Samples
Shielding effectiveness against neutron radiation	50 × 50 × 5	From 1 to 10
Compressive strength	10 × 10 ×10	3
Microstructure	4 × 4 × 1	9

**Table 4:** Neutron radiation shielding efficiency expressed as half value layer *HVL.*

*HVL* (cm)					
*M*	*MPCC1*	*MPCC2*	*MF1*	*MF2*	*MF3*
7.27	7.08	7.62	7.22	7.86	8.52

**Table 5:** The results of compressive strength *fc.*

	*Fc* (MPa)					
	*M*	*MPCC1*	*MPCC2*	*MF1*	*MF2*	*MF3*
$\bar{X}$(fc) (MPa)	42.7	43.4	51.3	44.3	52.0	25.7
*CV*(fc) (%)	12.0	3.6	4.5	5.1	2.8	11.1
**Legend:**	$\bar{X}$ – mean value		*CV* – coefficient of variation			

**Table 6:** Stereological parameters calculated using the computer image analysis method.

Parameter		Definition					
		*M*	*MPCC1*	*MPCC2*	*MF1*	*MF2*	*MF3*
*V_V_*	$\bar{X}$ (%)	5.23	0.96	2.15	1.71	1.96	5.69
	*CV* (%)	12.17	13.30	24.41	16.71	14.10	19.50
*S_V_*	$\bar{X}$ (mm^–1^)	0.87	0.22	0.34	0.27	0.30	0.48
	*CV* (%)	8.06	5.37	8.31	8.28	11.36	12.46
$\bar{L}$	$\bar{X}$ (mm)	1.08	1.53	1.67	1.75	1.70	2.04
	*CV* (%)	7.36	8.85	13.86	10.40	9.33	8.10
*CV(d_2V_)*	$\bar{X}$ (%)	34.22	36.96	36.39	37.97	36.45	35.69

**Table 7:** Results of Student’s *t*-test comparing the *HVL* of the reference concrete with those of dispersion-modified concretes and fiber-modified concretes (statistically not significant differences between *HVL* values were marked with grey).

Polymer Form	*t* Statistic	*p* Value
Dispersion (*MPCC1* and *MPCC2*)	–0.17	0.8921
Fibers (*MF1*, *MF2*, and *MF3*)	–0.79	0.5100

**Table 8:** Results of Student’s *t*-test comparing the *fc* of the reference concrete with those of dispersion-modified concretes and fiber-modified concretes (statistically not significant differences between *fc* values were marked with grey).

Polymer Form	*t* Statistic	*p* Value
Dispersion (*MPCC1* and *MPCC2*)	4.50	0.0028
Fibers (*MF1*, *MF2*, and *MF3*)	2.76	0.0202

## Appendix A

**Table A1:** Results of a comparison of the stereological parameters of *M* concrete with parameters of *MPCC1*, *MPCC2*, *MF1*, *MF2*, and *MF3* concretes using Student’s *t*-test: *t* statistics and *p* values (statistically not significant differences between parameters were marked with grey).

Concrete/Parameters		*M*			
		*V_V_* (%)	*S_V_* (mm^−1^)	$\bar{L}$	*CV(d_2V_)* (%)
***MPCC1***	***V_V_* (%)**	*t* = 18.62 *p* < 0.00001	-	-	-
	***S_V_* (mm^−1^)**	-	*t* = 25.85 *p* = 0.00001	-	-
	$\bar{L}$ **(mm)**	-	-	*t* = −7.99 *p* < 0.00001	-
	***CV(d_2V_)* (%)**	-	-	-	*t* = −3.98 *p* = 0.00109
***MPCC2***	***V_V_* (%)**	*t* = 10.59 *p* < 0.00001	-	-	-
	***S_V_* (mm^−1^)**	-	*t* = 19.90 *p* < 0.00001	-	-
	$\bar{L}$ **(mm)**	-	-	*t* = −6.75 *p* < 0.00001	-
	***CV(d_2V_)* (%)**	-	-	-	*t* = −2.65 *p* = 0.01737
***MF1***	***V_V_* (%)**	*t* = 14.29 *p* < 0.00001	-	-	-
	***S_V_* (mm^−1^)**	-	*t* = 23.12 *p* < 0.00001	-	-
	$\bar{L}$ **(mm)**	-	-	*t* = −9.47 *p* < 0.00001	-
	***CV(d_2V_)* (%)**	-	-	-	*t* = −3.73 *p* = 0.00181
***MF2***	***V_V_* (%)**	*t* = 13.34 *p* < 0.00001	-	-	-
	***S_V_* (mm^−1^)**	-	*t* = 20.57 *p* < 0.00001	-	-
	$\bar{L}$ **(mm)**	-	-	*t* = −9.77 *p* < 0.00001	-
	***CV(d_2V_)* (%)**	-	-	-	*t* = −2.26 *p* = 0.03779
***MF3***	***V_V_* (%)**	*t* = −1.02 *p* = 0.32312	-	-	-
	***S_V_* (mm^−1^)**	-	*t* = 11.74 *p* < 0.00001	-	-
	$\bar{L}$ **(mm)**	-	-	*t* = −14.73 *p* < 0.00001	-
	***CV(d_2V_)* (%)**	-	-	-	*t* = −2.14 *p* = 0.04817
